# Supplementary material for: Radiofrequency Lesion in the Atrial Wall: How Variable Is It? 9.4 Tesla MRI Analysis of Radiofrequency Lesion Volume in a Swine Model
Source: J Clin Med. 2024 Aug 30;13(17):5153. doi: 10.3390/jcm13175153 (PMC11396762; doi:10.3390/jcm13175153)

Supplementary tables and figures

Supplementary Table S1. Extracardiac lesions in each group.

Supplementary Table S2. Predictors of extracardiac lesions from the univariate logistic regression analysis.

Supplementary Figure S1. Distribution of lesion volumes based on the presence of extracardiac lesions between groups.

Tables

Supplementary Table S1. Extracardiac lesions in each group.

	Low power (20W)<br>*n = 26*	High power (50W)<br>*n = 28*	*p* Value
Extracardiac lesions, *n (%)*	14 (53.8)	18 (64.3)	0.613
Right upper lung lobe, *n*	3	1	
Right medium lung lobe, *n*	3	4	
Right inferior lung lobe, *n*	1	0	
Left upper lung lobe, *n*	1	2	
Left inferior lung lobe, *n*	0	2	
Pulmonary arterial trunk, *n*	1	2	
Trachea, *n*	1	1	
Aorta, *n*	0	1	
Inferior vena cava, *n*	0	1	
Others, *n*	4	4	

Supplementary Table S2. Predictors of extracardiac lesions from the univariate logistic regression analysis.

	Univariate analysis	
	**OR (95% CI)**	***p Value***
RAs	3.000 (0.879 – 10.244)	0.080
RAi	0.478 (0.141 – 1.624)	0.237
LAs	0.697 (0.213 – 2.284)	0.551
LAi	1.000 (0.310 – 3.226)	1.000
Flat surface of the ablation	0.556 (0.190 – 1.621)	0.282
Trabecular surface of ablation	3.667 (1.141 – 11.787)	**0.029**
Lesion volume (cm^3)	3.971 (0.112 – 140.265)	0.448
Lesion depth (mm)	0.988 (0.643 – 1.517)	0.954
Sample thickness (mm)	1.135 (0.833 – 1.546)	0.422

RAs: right atrium near the superior vena cava; RAi: right atrium near the inferior vena cava; LAs: superior part of the left atrium; LAi: inferior part of the left atrium; OR, odds ratio; CI, confidence interval.

Figure

Supplementary Figure S1. Distribution of lesion volumes based on the presence of extracardiac lesions between groups. *LE, low energy; HE, high energy*.

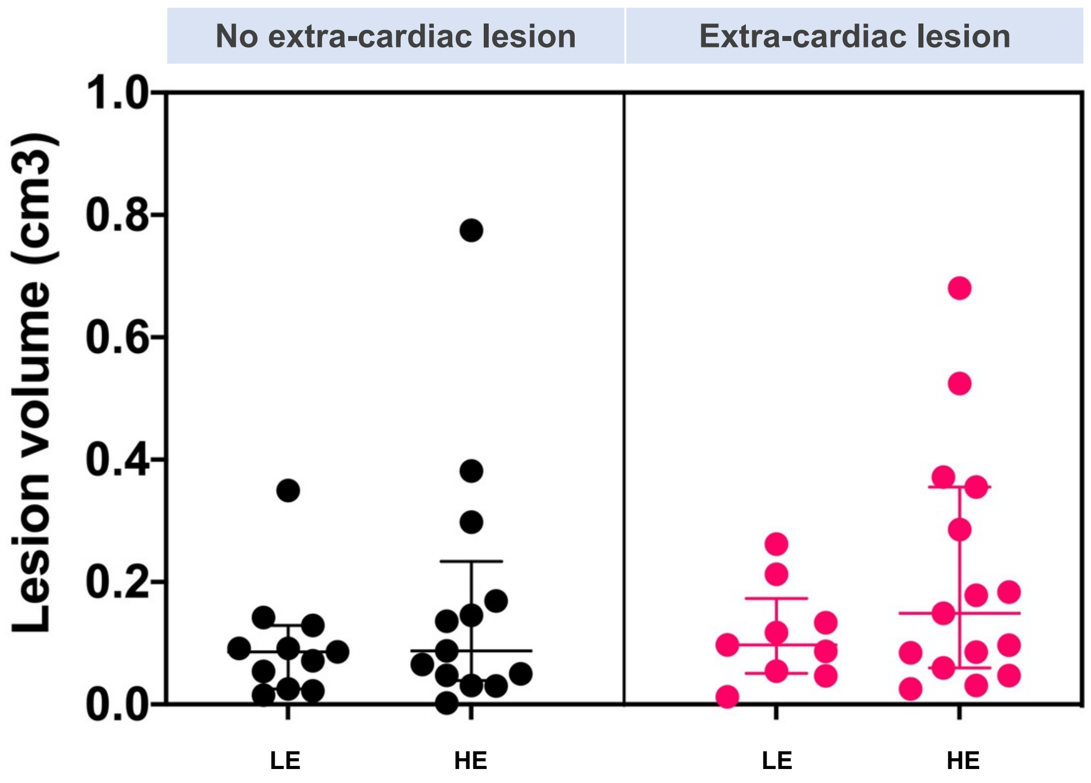

Supplement: Supplementary file 1 [file jcm-13-05153-s001.zip › jcm-3146119-supplementary.pdf]
